# The Role of Alpha-2 Agonists for Attention Deficit Hyperactivity Disorder in Children: A Review

**DOI:** 10.3390/neurolint15020043

**Published:** 2023-05-22

**Authors:** Elisa E. Neuchat, Brooke E. Bocklud, Kali Kingsley, William T. Barham, Patrick M. Luther, Shahab Ahmadzadeh, Sahar Shekoohi, Elyse M. Cornett, Alan D. Kaye

**Affiliations:** 1 School of Medicine, Louisiana State University Health Sciences Center at Shreveport, Shreveport, LA 71103, USA; 2 School of Medicine, Louisiana State University Health Sciences Center at New Orleans, New Orleans, LA 70112, USA; 3 Department of Anesthesiology, Louisiana State University Health Sciences Center at Shreveport, 1501 Kings Highway, Shreveport, LA 71103, USA; 4 Department of Pharmacology, Toxicology, and Neurosciences, Louisiana State University Health Sciences Center at Shreveport, 1501 Kings Highway, Shreveport, LA 71103, USA

**Keywords:** Alpha-2 agonist, attention deficit hyperactivity disorder (ADHD), neurodevelopmental disorder, clonidine, guanfacine

## Abstract

Introduction: Attention Deficit Hyperactivity Disorder (ADHD) is one of the most common neurodevelopmental disorders, characterized by the Diagnostic and Statistical Manual of Mental Disorders, Fifth Edition (DSM-5), which is marked by symptoms such as inappropriate levels of inattention, hyperactivity, and impulsivity that can affect academic, social, and personal functioning in children and adolescents. This review summarizes clinical trials demonstrating the effectiveness of Alpha-2 agonists in reducing symptoms of inattention, hyperactivity, and impulsivity in children with ADHD. Studies were identified through a systematic search of PubMed and Cochrane databases. However, these medications’ long-term safety and efficacy remain uncertain, with a lack of data on their effects on growth, cardiovascular function, and other adverse events. Further studies are required to determine these medications’ optimal dose and treatment duration. Methods: Medications that target the noradrenergic system, such as Alpha-2 agonists, have been increasingly used as a treatment option for ADHD, with guanfacine and clonidine being two of the most commonly used medications. They function by selectively targeting Alpha-2 adrenergic receptors in the brain leading to improved attention and reduced hyperactivity and impulsivity symptoms in children with ADHD. Results: Clinical trials have demonstrated the effectiveness of Alpha-2 agonists in treating ADHD in children by reducing symptoms of inattention, hyperactivity, and impulsivity. However, these medications’ long-term safety and efficacy still need to be completely understood. Due to a lack of information on the effects of Alpha-2 agonists on growth, cardiovascular function, and other long-term adverse events, more studies must investigate the optimal dose and treatment duration for these medications. Conclusions: Despite these concerns, Alpha-2 agonists remain a valuable treatment option for ADHD in children, especially those unable to tolerate stimulant medications or who have coexisting conditions such as tic disorders. Future research should continue to explore the safety and efficacy of Alpha-2 agonists in the long term. In conclusion, Alpha-2 agonists show promise as a treatment for ADHD in children; however, the safety and efficacy of these drugs in the long term are not yet completely understood. Additional studies are required to investigate the optimal dose and treatment duration for these medications in their use as a treatment for this debilitating disease.

## 1. Introduction

Attention deficit hyperactivity disorder (ADHD) is one of the most common mental diseases affecting children and adolescents. The main features of ADHD include difficulty with attention and concentration, impulsivity, and hyperactivity. These symptoms can significantly impact a child’s academic and social functioning [1]. ADHD is diagnosed in childhood and can persist into adulthood. The current standard of care for ADHD includes using stimulant medications, such as methylphenidate and amphetamines, and non-stimulant medications, such as atomoxetine [2]. These medications are effective in decreasing symptoms of ADHD, but they can also have significant side effects [3]. Alpha-2 agonists, such as guanfacine and clonidine, have been studied as a treatment option for ADHD in children. These medications selectively target the Alpha-2 adrenergic receptors in the brain, which can enhance attention and decrease impulsivity and hyperactivity symptoms in children with ADHD. The mechanism of action of Alpha-2 agonists is different from the traditional medications used for ADHD, and it is thought to be more specific and therefore has fewer side effects [3]. Research has shown that Alpha-2 agonists can be effective in decreasing symptoms of ADHD in children. Many studies assessed the efficacy and safety of these medications [4]. For example, a randomized, double-blind, placebo-controlled study conducted in 2013 found that guanfacine extended-release (XR) significantly improved symptoms of ADHD in children aged 6–12 years. Another study conducted in 2018 found that clonidine effectively reduced ADHD symptoms in children aged 6–12 years with comorbid tic disorders [5]. In addition to their efficacy, Alpha-2 agonists may also have advantages over traditional medications for ADHD in terms of their side effect profiles. Stimulant medications, such as methylphenidate and amphetamines, can cause side effects, including insomnia, decreased appetite, and weight loss. Non-stimulant medications like atomoxetine can cause side effects such as nausea, vomiting, and liver toxicity. In contrast, Alpha-2 agonists are generally well-tolerated and have been associated with fewer side effects [6].

Despite the potential benefits of Alpha-2 agonists, their use has some limitations. One of the major restrictions is that they may not be as functional as stimulant medications in treating the core symptoms of ADHD, particularly hyperactivity. Another limitation is that they may cause sedation, which can interfere with academic and social functioning. Also, Alpha-2 agonists may have cardiovascular side effects including reduced blood pressure and heart rate, which require monitoring [7].

Recent studies have explained that ADHD is not a homogeneous disorder, and there is considerable variability in treatment response among individuals [8]. Genetic variations, comorbidities, and environmental influences may play a role in treatment outcomes [9]. Therefore, identifying predictors of treatment response and individualizing treatment plans for each patient may improve treatment efficacy and reduce adverse effects. Additionally, the optimal dosing and duration of treatment with Alpha-2 agonists in children with ADHD remain unclear. Future studies should address these knowledge gaps to provide more personalized and effective treatment for children with ADHD. By investigating the efficacy and safety of Alpha-2 agonists and identifying factors that influence treatment response, we can advance our understanding of this complex disorder and provide better care for children and adolescents with ADHD.

Overall, Alpha-2 agonists have emerged as a promising treatment option for ADHD in children. They have a different action mechanism than traditional medications and may have fewer side effects. However, more research is required to investigate their long-term efficacy and safety, particularly in comparison to stimulant medications. Clinicians should consider each child’s needs and characteristics when making treatment decisions for ADHD, and Alpha-2 agonists may be a useful addition to the treatment arsenal for some children with ADHD.

The focus of this paper is to go through the current evidence regarding the use of Alpha-2 agonists for ADHD in children and adolescents. Specifically, we aim to assess the efficacy and safety of these medications, compare them to traditional medications such as stimulants and non-stimulants, and identify predictors of treatment response. Additionally, we aim to provide recommendations for clinicians regarding the use of Alpha-2 agonists in the treatment of ADHD in children.

We are revising the topic of Alpha-2 agonists because they have emerged as a promising treatment option for ADHD in children, and there is a requirement for a comprehensive review of the current evidence regarding their use. In addition, with the variability in treatment response among individuals and the potential for adverse effects related to traditional medications, it is important to explore alternative treatment options, such as Alpha-2 agonists to provide personalized and effective care for children with ADHD.

## 2. Current Understanding of the Use of Alpha-2 Agonists as a Treatment Option for ADHD in Children

Alpha-2 agonists stimulate alpha-2 receptors, most predominantly in the prefrontal cortex. The most used agents for ADHD are clonidine and guanfacine. Clonidine stimulates Alpha-2A, Alpha-2B, and Alpha2C, whereas guanfacine stimulates the Alpha-2A receptors in the prefrontal cortex. These actions on both the pre-and post-synaptic membrane alter arousal and cognitive processes that are implicated in ADHD. The most prominent side effect of both drugs is sedation and orthostatic hypotension. Sudden discontinuation of these drugs could also result in rebound hypertension. However, it has milder side effects than clonidine due to the longer duration of action of guanfacine’s longer duration of action [10].

Alpha-2 agonists are second-line agents to stimulants. They were originally administered simultaneously to extend the duration of action of stimulants. Some data showed that doing this allowed for equal efficacy while using a smaller dose of stimulant. Also, patients with ADHD are prone to sleep disorders that can be worsened with use of stimulants. Due to the sedative side effects of Alpha-2 agonists, especially clonidine, they can be advantageous in patients experiencing insomnia. Now that there are long-action formulations of stimulants, this is no longer indicated unless additional therapy is required for complex ADHD. Examples are when a patient suffers from comorbid conditions such as Tourette syndrome, oppositional defiant disorder, or aggressive/impulsive behavior [11]. These comorbid conditions often respond poorly to stimulants alone. In addition, research has shown that patients can have worsening core symptoms of autism when treating their ADHD with stimulants [10].

Monotherapy of Alpha-2agonists for ADHD is currently being researched. It is currently thought that they are effective in treating the hyperactivity and impulsivity of ADHD but not in improving inattention. Also, current formulations do not maintain adequate plasma concentrations without causing significant side effects. There are currently phase 3 trials underway on sustained-release formulations of clonidine, but Alpha-2 agonist monotherapy for ADHD is considered off-label [11].

Recent studies have also investigated the potential use of Alpha-2 agonists as a monotherapy for ADHD in children [12,13]. However, studies in this field are still in their early steps, and more studies are needed to completely understand the effectiveness of these medications when used alone. It is believed that Alpha-2 agonists may be effective in treating hyperactivity and impulsivity symptoms but may not be as effective in improving inattention symptoms compared to stimulant medications. Additionally, current formulations of Alpha-2 agonists may not maintain adequate plasma concentrations without causing significant side effects, making it challenging to achieve optimal treatment outcomes [12]. Nevertheless, sustained-release formulations of clonidine are currently in phase 3 trials, which may provide a more viable option for monotherapy in the future [12]. Despite the current limitations, the potential benefits of Alpha-2 agonists, such as fewer side effects and potential effectiveness in treating specific symptoms, make them an interesting area of study for treating ADHD in children. Continued research and exploration of these medications as a monotherapy may provide additional treatment options for children with ADHD.

## 3. Safety and Effectiveness of Alpha-2 Agonist drugs

Guanabenz, guanfacine, clonidine, tizanidine, medetomidine, and dexmedetomidine are all α-2 agonists that vary in their potency and affinities for the various α-2 receptor subtypes. Clonidine, tizanidine, and dexmedetomidine are metabolized in the liver have received the greatest clinical use [14]. Clonidine and guanfacine are drugs used to treat attention deficit/hyperactivity disorder in children and adolescents. These drugs work via agonism of the α2 receptors which decrease the firing rate of presynaptic neurons and release norepinephrine into the prefrontal cortex. In turn, this opens potassium channels and decreases cyclic adenosine monophosphatase levels. Clonidine is believed to primarly improve impulsivity and hyperactivity in ADHD through this mechanism [15]. Clonidine has both central and peripheral actions. In the central nervous system, stimulation of the α2-adrenergic receptors increase vagal tone leading to a reflex increase in parasympathetic activity and inhibition of sympathetic outflow. This inhibition of the efferent sympathetic pathway results in a decrease in the vascular tone of the heart, kidneys and peripheral vasculature and a reduction in peripheral resistance, which is the primary mechanism by which clonidine regulates blood pressure [15].

Clinical trials have demonstrated that guanfacine, a preferential α_2A_-adrenoceptor agonist, is an effective treatment for ADHD treatment in children and adolescents. These trials primarily focused on the combined ADHD subgroup, which includes both hyperactive/impulsive and inattentive symptoms [16]. Nevertheless, it has been observed that guanfacine is not as effective in treating the inattentive subtype of ADHD compared to other subtypes. In children receiving guanfacine treatment, response rates of 50–60% have been reported, which is comparable to other non-stimulant drugs in terms of efficacy [16]. Adverse events associated with guanfacine include hypotension, bradycardia, and in some cases, syncope, drowsiness, fatigue, sedation, upper abdominal pain, dry mouth, nausea, and dizziness, as reported by studies [17]. Unlike other ADHD medications, guanfacine has been found to cause moderate weight gain. A study was conducted to investigate the long-term safety and effectiveness of guanfacine extended-release (GXR) in treating adults with ADHD [18]. The study revealed no significant safety concerns associated with the long-term use of GXR to ADHD adults. The most frequently reported treatment-emergent adverse events (TEAEs), occurring in 10% of patients, were drowsiness, thirst, nasopharyngitis, decreased blood pressure, bradycardia, malaise, constipation, and postural dizziness. Patients treated with GXR showed significant improvements in ADHD symptoms, executive functioning and quality of life after long term treatment, compared to baseline [18].

Over 13 weeks, a randomized, placebo-controlled trial was performed to determine the safety and efficacy of guanfacine extended release (GXR) in adolescents with ADHD. Most participants received optimal doses of 3, 4, 5, or 6 mg, with 46.5% receiving optimal dose in excess of the currently permitted maximum of 4 mg. Most treatment-emergent adverse events were mild to moderate in severity, with sedation-related events being the most frequently reported [19].

## 4. Optimal Dosing and Duration of Treatment for These Medications

Clonidine and guanfacine are Alpha-2 agonist drugs, that can be administered through oral, transdermal, intravenous, or epidural routes [14]. Clonidine’s oral administration is rapidly absorbed within 3 to 5 h, while the extended-release formula has a slower absorption rate, with peak absorption occurring within 4 to 7 h. The transdermal form of clonidine is absorbed at a constant rate for 7 days, with an initial Tmax of 2 to 3 days [15]. The immediate-release formulation of clonidine starts showing effects within 30 to 60 min, with peak effects observed within 2 to 4 h. The effectiveness can last up to 8 h after administration, and similarly, the transdermal form may last for 8 h after discontinuation [15].

For clonidine’s oral dosage form (extended-release tablets), the initial dosing for teenagers and children aged six years and older is 0.1 milligram (mg) once a day, given at bedtime [20].

For guanfacine’s oral dosage form (extended-release tablets), the initial dosing for adults and children aged six years and older is 1 mg once a day, either in the morning or evening, at the same time each day. The doctor may adjust the dose using increasing increments (not exceeding 1 mg/wk). The recommended target dose range, based on clinical response and tolerability, is 0.05–0.12 mg/kg/day PO initially. For children aged 6–12 years, doses >4 mg/day have not been evaluated, while for those aged 13–17 years, doses >7 mg/day have not been evaluated [21]. In a randomized placebo-controlled study of adolescents, a total of 314 participants were randomized, with most participants receiving optimal doses of 3, 4, 5, or 6 mg (30 [22.9%], 26 [19.8%], 27 [20.6%], or 24 [18.3%] participants, respectively). However, 46.5% of participants received an optimal dose above the currently approved maximum dose limit of 4 mg [19].

Optimal dosing and duration of treatment for Alpha-2 agonists, such as clonidine and guanfacine, must be individualized to the patient’s needs and response. While there are general dosing guidelines for these medications, the optimal dose may vary depending on the patient’s age, weight, and comorbidities. For example, renal or hepatic impairment patients may require lower doses of these medications to avoid potential toxicity [16]. Additionally, the duration of treatment may vary depending on the patient’s reaction to therapy and the severity of their symptoms. Some patients may require long-term treatment, while others may only need short-term treatment to manage acute symptoms. Hence, it is essential for healthcare providers to regularly monitor patients receiving Alpha-2 agonists to assess treatment response and evaluate any adverse effects. Moreover, dose titration is necessary to achieve the desired therapeutic effect while minimizing adverse effects. Patients should start at the lowest possible dose and increase gradually, under medical supervision, until the optimal dose is reached. The optimal dose is the lowest effective dose that provides clinical benefit without causing significant adverse effects. When discontinuing treatment, patients should be gradually tapered off the medication to avoid rebound hypertension or other adverse effects [17]. Additionally, treatment duration and dosage must be evaluated on a case-by-case basis, and patients must receive close medical follow-ups to ensure that they receive the best possible care for their ADHD symptoms.

Patients may require different dosages and treatment durations to attain the desired therapeutic effect. In addition, these medications can cause adverse effects, so patients should be closely monitored to make sure that the profits of treatment outweigh the risks.

## 5. Clinical Studies on the Efficacy of Alpha 2 Agonists in Children with ADHD

Current clinical practice guidelines for treating ADHD in elementary school-aged children rate stimulants, atomoxetine, extended-release guanfacine, and extended-release clonidine as most effective (in that order), with an effect size of 1 for stimulants and 0.7 for non-stimulants [22]. In meta-analyses comparing stimulants and non-stimulants, the effect size of stimulants is larger for treating ADHD in children [23]. Decreased dynamic functional connectivity has been demonstrated in children treated with long-acting amphetamine lisdexamphetamine, suggesting their therapeutic effect is mediated through thalamic neural circuits [24]. Another amphetamine, methylphenidate, has been used to increase executive functioning in children with ADHD [25]. Additionally, methamphetamine salts have been used in children aged 6–17 with ADHD. However, their use is not superior to placebo and may be accompanied by side effects that plague the use of amphetamines and other stimulants, such as appetite loss, abdominal pain, headaches, and sleep disturbance [26].

Because not all children are candidates for treatment with the first-line choice stimulants, and some may not be fully treated with stimulant monotherapy, alpha 2 agonists represent a potentially useful alternative monotherapy or adjunctive therapy to traditional stimulants. Also, alpha 2 agonists, such as guanfacine and clonidine, may have a more favorable side effect profile [27]. Side effects specific to alpha 2 agonists include somnolence, dry mouth, bradycardia, and rebound hypotension [28].

In randomized control trials examining alpha 2 agonist guanfacine as a monotherapy in children with ADHD, somnolence, headache, and fatigue were observed as adverse effects in a cohort consisting of 6–17-year-old patients [29]. This study examined secondary variables in the domain of family functioning and school functioning. The onset of treatment action was more rapid than for the control arm of patients treated with atomoxetine [29]. This is concordant with meta-analyses comparing guanfacine versus atomoxetine in children with oppositional symptoms; across one guanfacine trial and six atomoxetine trials, guanfacine was associated with a greater reduction of symptoms, although these trials only compared short-term usage [30]. In 6–12-year-old patients, both morning and evening guanfacine XR monotherapy was superior to placebo [31]. In guanfacine’s use as adjunctive therapy with stimulant dexmethylphenidate, increased benefits in symptom management in 7–14-year-old patients were observed over monotherapy with either drug, without an increase in safety concerns [32]. It may be that dual modulation of the dopaminergic and adrenergic axes increases symptom management, but the effect size of dual therapy versus monotherapies reported in this trial was modest. Electroencephalography profiles of boys aged 7–14 on combination treatment have demonstrated that decreased neural activity in the midoccipital region, which plays a role in visuo-spatial attention, may mediate this effect [33]. An open-label trial comparing methylphenidate or amphetamine in combination with guanfacine XR in monotherapy resistant male and female 6–17-year-old patients found upper abdominal pain (25.3%), fatigue (24.0%), irritability (22.7%), headache (20.0%), and somnolence (18.7%) as adverse effects, although most of these events were characterized as mild and did not lead to treatment discontinuation [34].

Guanfacine may have unique utility in children with ADHD with other comorbidities. In a randomized control trial of children with autism, Guanfacine was superior to a placebo, and showed efficacy in reducing hyperactivity and impulsiveness, with lower discontinuation rates than traditional stimulant methylphenidate [35]. Guanfacine has also been shown to reduce tics in children with comorbid ADHD [35]. In children 6–12 years old with oppositional defiant disorder, guanfacine XR at 1–4 mg/day for nine weeks was superior to placebo in reducing ADHD Rating Scale score, with a high correlation (r = 0.74) of reduction in oppositional symptoms and ADHD symptoms [36]. In 6–18-year-old children and adolescents, symptoms of ADHD, stress, anxiety, and current trauma symptoms were decreased in children taking guanfacine XR at 1–4 mg/day [37]. Like the findings with tics and hyperactivity-related disorders, it may be that guanfacine is facilitating higher brain center control over reactive, external stimulus-driven pathways [38].

Clonidine, another centrally acting alpha 2 agonist, has been used off-label for years in treating ADHD [39]. Although Clonidine has efficacy in reducing ADHD symptomatology, it is not favored for long-term treatment due to the risk of hypotension and a less favorable pharmacokinetic profile [40]. Switching patients from Clonidine IR to Guanfacine XR is one option for managing ADHD symptomatology, with tapering of doses slowly to prevent rebound hypertension when transitioning pharmacological treatments [41]. This dosage titration process may take up to one week [41]. One proposed mechanism for the increased hypotensive effects of Clonidine relative to other alpha 2 agonists is Clonidine’s affinity for imidazole receptors in the rostral ventrolateral medulla [42]. In an 8-week double-blinded randomized controlled trial of children 7–12 years old, Clonidine was inferior to methylphenidate in ADHD symptom management, mirroring the relative inferiority of Guanfacine relative to traditional stimulants in this population [43]. Clonidine may be effective in other populations with behavioral disturbances, such as autism spectrum disorder, but there is a lack of literature on Clonidine’s use in this subgroup [44]. Clonidine combination therapy with stimulants has also shown superiority to stimulant monotherapy in children 6–14 years old. However, hypotension remains a prominent unwanted side effect in this cohort (Table 1 and Table 2) [45].

## 6. Screening and Selection of Papers for Review

The screening and selection process for this paper, “The Role of Alpha-2 agonists for Attention Deficit Hyperactivity Disorder in Children” involved a comprehensive search of electronic databases, including PubMed and Cochrane. The search was restricted to papers published in English between 2000 and 2023. The initial search yielded around 300 articles of which hundred were duplicates. The remaining articles were screened according to their topics and abstracts, and around 50 were deemed potentially relevant.

The selected articles included randomized controlled trials, observational studies, meta-analyses, and systematic reviews that investigated the use of Alpha-2 agonists in treating ADHD in children.

Overall, the review aimed to provide a comprehensive and up-to-date summary of the role of Alpha-2 agonists in treating ADHD in children according to the best available evidence from the literature.

## 7. Effects of Alpha-2 Agonists on Neuropsychological Profile, Emotional/Behavioral Problems, and Parental Stress in Children with ADHD

Several studies have shown that children with ADHD exhibit specific deficiencies in cognitive and emotional/behavioral domains compared to typically developing children. In particular, deficits in attention, executive function, and working memory have been consistently reported in the literature (Barkley, 2014; Loe & Feldman, 2007) [48,49] Emotional and behavioral problems, such as hyperactivity, impulsivity, oppositional behavior, and social difficulties, are common in children with ADHD (Barkley, 2014; Johnston & Mash, 2001) [48,50].

The use of Alpha-2 agonists, such as guanfacine and clonidine, effectively reduces symptoms of inattention, hyperactivity, and impulsivity in children with ADHD (Connor et al., 2013 [51]; Childress et al., 2018) [26]. Additionally, there is evidence that Alpha-2 agonists may improve cognitive functions such as working memory and attentional control (Arnsten & Pliszka, 2011 [52]; Schmaal et al., 2017 [53]). One study found that guanfacine improved working memory and cognitive flexibility in children with ADHD, while another showed that clonidine improved attentional control (Franke et al., 2017 [54]; Stein et al., 1995 [55]).

Furthermore, parental stress is a significant issue in families of children with ADHD (Giallo et al., 2015 [56]). The problems of managing a child with ADHD, such as difficulty with routines, behavior problems, and academic struggles, can significantly strain parents and family life. However, the use of Alpha-2 agonists may have a positive impact on parental stress levels. A study by Waxmonsky et al. (2013) [57] found that treatment with extended-release guanfacine reduced parental stress levels in families of children with ADHD compared to placebo treatment. Similarly, another study showed that treatment with clonidine improved parent-rated quality of life in families of children with ADHD (Scahill et al., 2001) [47].

In summary, the use of Alpha-2 agonists in the treatment of ADHD in children may not only reduce symptoms of inattention, hyperactivity, and impulsivity but may also improve cognitive functioning and reduce parental stress levels. However, further research is essential to assess better the mechanisms underlying these effects and to identify optimal dose and treatment durations.

## 8. Discussion

Attention deficit hyperactivity disorder (ADHD) is a common neurodevelopmental disease affecting children and adults and it can interfere with daily functioning and quality of life. The causes of ADHD are not fully understood, but genetic, environmental, and neurobiological factors are believed to contribute to its development [58].

Managing ADHD can be challenging, particularly in children who may struggle with academic and social demands in addition to their symptoms. The primary treatments for ADHD are medication and behavioral therapies and Alpha-2 agonists have emerged as a promising option for treating ADHD in children [1]. Guanfacine and clonidine, are two Alpha-2 agonists confirmed by the US Food and Drug Administration (FDA) for treating ADHD in children, and they have been found effective in reducing symptoms of ADHD, particularly in the areas of hyperactivity, impulsivity, and inattention, as well as improving overall functioning, including academic and social outcome [59]. One of the advantages of Alpha-2 agonists over other ADHD medications, such as stimulants, is their lower potential for abuse and addiction. However, Alpha-2 agonists can cause side effects, including dizziness, fatigue, headaches, and interactions with other medications that need to be carefully considered [14].

## 9. Conclusions

In summary, Alpha-2 agonists are a promising addition to the treatment options for ADHD in children. While they have potential side effects and interactions, they offer healthcare professionals and parents/caregivers another option for managing ADHD in children. By working closely with healthcare providers and carefully monitoring their children’s treatment, parents and caregivers can help ensure the best outcomes for children with ADHD.

## Figures and Tables

**Table 1:** Alpha 2 Agonist Central Nervous System Mechanism of Action.

Study	Neural Correlate
Michelini et al., 2023 [33] *Randomized Control Trial*	Combination treatment with guanfacine and D-methylphenidate in children with ADHD decreases EEG power over most frequency bands and task phases in cingulate and primary visual cortex.
Mizuno et al., 2022 [46] *Randomized Control Trial*	Methylphenidate improves sustained attention and increases dwell state in cross-network pathways modulating salience, the default mode network, and the frontoparietal network.
Rosenau et al., 2021 [25] *Randomized Control Trial*	Long-term use of stimulant methylphenidate in children and adolescents aged 8–18 improves performance on working memory tasks relative to the control group who discontinued psychostimulant treatment.
Wang et al., 2022 [24] *Randomized Control Trial*	Reduced dynamic thalamic functional connectivity mediates the therapeutic effect of long-acting psychostimulants amphetamine and lisdexamphetamine in ADHD patients 6–25 years old.

**Table 2:** Alpha 2 Agonists in Children with ADHD and Selected Co-Morbidities.

Study	Co-Morbidities
Politte et al., 2018 [35] *Randomized Control Trial*	In children with Autism Spectrum Disorder and ADHD aged 5–14 years old, extended released Guanfacine was superior to placebo in reducing oppositional behavior and repetitive behavior, but was not superior to placebo in improving sleep habits or reducing anxiety.
Connor et al., 2013 [37] *Open Label Pilot Study*	In children aged 6–18 years old with current traumatic stress symptoms, re-experiencing, avoidant, and overarousal symptoms were significantly improved with administration of Guanfacine, and these positive effects were accompanied by decreased ADHD symptomatology.
Scahill et al., 2001 [47] *Randomized Control Trial*	In children with comorbid ADHD and tic disorder, 8 weeks of Guanfacine treatment decreased tic severity by 31%, compared to a 0% decrease in tic severity in the placebo control group.
Connor et al., 2010 [36] *Randomized Control Trial*	In children with ADHD and comorbid oppositional symptoms, extended release Guanfacine over 9 weeks decreased ADHD symptoms on ADHD Rating Scale IV (ADHD-RS-IV) and parental rating of oppositional symptoms from baseline, and these decreases were highly correlated (r = 0.74).

## Data Availability

Not applicable. Data sharing is not applicable to this article as no datasets were generated or analyzed during the current study.
